# Jervell and Lange-Nielsen Syndrome Manifesting as Seizure-Like Episodes in Childhood

**DOI:** 10.7759/cureus.94011

**Published:** 2025-10-07

**Authors:** Ans Ahmad, Faraz Ahmad, Muhammad Masudul Hasan Nuri

**Affiliations:** 1 Cardiology, Tahir Heart Institute, Chenab Nagar, PAK

**Keywords:** arrhythmias, congenital long qt syndrome, jervell and lange-nielsen syndrome, sensorineural deafness, sudden cardiac death, syncope

## Abstract

Jervell and Lange-Nielsen syndrome (JLNS) is a rare autosomal recessive form of congenital long QT syndrome (LQTS) characterized by prolonged QT interval and congenital bilateral sensorineural deafness. We report the case of an eight-year-old boy with recurrent convulsions and syncope, unresponsive to antiseizure treatment. He had a history of congenital bilateral sensorineural deafness and the sudden death of a sister. His ECG revealed a markedly prolonged QT interval of 680 ms (corrected QT interval: 650 ms). A diagnosis of JLNS was made, and propranolol was initiated. The parents were counseled on avoiding emotional and physical triggers and referred for consideration of an implantable cardioverter-defibrillator. JLNS can mimic epilepsy caused by arrhythmia-induced cerebral hypoperfusion, leading to convulsions. Such episodes can be distinguished from epilepsy by their brief duration, syncope preceding convulsions, and a family history of LQTS or sudden unexplained death. This case highlights the need for a high index of suspicion in such cases, as early diagnosis through detailed history, ECG, and clinical evaluation, even without genetic confirmation, is lifesaving and essential to reduce the risk of sudden cardiac death.

## Introduction

Congenital long QT syndrome (LQTS) is a cardiac repolarization disorder marked by the prolongation of the QT interval. The most frequent type, known as Romano-Ward syndrome, involves an autosomal dominant inheritance pattern. Jervell and Lange-Nielsen syndrome (JLNS) is a rarer, more severe autosomal recessive variant, distinguished by the presence of both QT prolongation and congenital bilateral sensorineural deafness [1]. It was first reported by Norwegian physicians Anton Jervell and Fred Lange-Nielsen in 1957 in children with congenital deafness and recurrent episodes of syncope [2]. It is extremely rare, with a reported prevalence of less than one to six cases per 1,000,000 individuals [3,4].

The QT interval is usually prolonged beyond 500 ms, which predisposes these individuals to dangerous life-threatening arrhythmias and the risk of sudden cardiac death [5]. It is a genetic disorder caused by mutations in certain genes encoding cardiac ion channels. JLNS most commonly results from mutations in the KCNQ1 or KCNE1 genes, which encode potassium channel subunits critical for cardiac repolarization. Early recognition is crucial, as appropriate treatment with beta-blockers and consideration of implantable cardioverter-defibrillators (ICDs) can be helpful in the prevention of sudden deaths [6].

## Case presentation

An eight-year-old boy presented with generalized tonic-clonic convulsions followed by syncope. These episodes were often triggered by emotional stress, such as laughter or crying, strenuous physical activity, or fear. Each episode lasted a few seconds and was followed by rapid recovery; however, according to the parents, if an episode lasted longer, the child seemed slightly drowsy afterward. His parents reported that these events started when he was around seven to eight months old. Despite being on various antiseizure drugs, the convulsions and fainting episodes continued. He had also been diagnosed with sensorineural deafness since birth. However, he achieved normal developmental milestones and had no evidence of cognitive impairment. A significant family history was also present: his younger sister experienced similar convulsions, fainting, and congenital deafness, and she died at the age of two and a half years after a similar episode.

**Figure 1:**
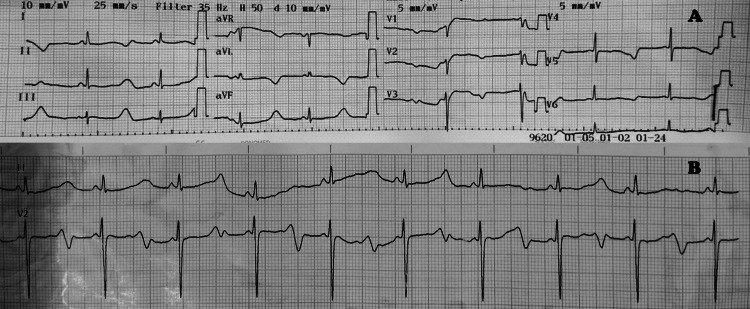
(A) Standard 12-lead ECG showing markedly prolonged QT interval of 680 ms and QTc of 650 ms. (B) Long leads highlighting the prolonged QT interval. QTc, corrected QT interval

On examination, he had a heart rate of 56 beats per minute. Physical and systemic examinations were otherwise normal. Neurological tests, including electroencephalogram and brain imaging, were normal, making a primary neurological cause less likely. Considering the clinical presentation and family history, a congenital heart problem was suspected. His ECG showed a significantly prolonged QT interval of 680 ms and a corrected QT interval (QTc) of 650 ms (Figure 1).

Echocardiography revealed normal heart chambers and valves without structural issues. Although genetic testing was unavailable due to cost and limited local facilities, the combination of sensorineural deafness, family history, and notably prolonged QTc strongly indicated JLNS. His Schwartz score, used to assess the likelihood of LQTS, was also high, with a score above six.

He was started on propranolol at a dose of 2 mg/kg/day, and the family was advised to avoid known emotional and physical triggers. They were also counseled on the importance of strict medication adherence and avoiding drugs that prolong the QT interval (Table 1).

**Table 1:** Drugs known to prolong the QT interval

Drug class	Examples
Antipsychotics	Chlorpromazine, haloperidol, droperidol, quetiapine, olanzapine, amisulpride, and thioridazine
Type IA antiarrhythmics	Quinidine, procainamide, and disopyramide
Type IC antiarrhythmics	Flecainide and encainide
Class III antiarrhythmics	Sotalol and amiodarone
Tricyclic antidepressants	Amitriptyline, doxepin, imipramine, nortriptyline, and desipramine
Other antidepressants	Mianserin, citalopram, escitalopram, venlafaxine, bupropion, and moclobemide
Antihistamines	Diphenhydramine, astemizole, loratadine, and terfenadine
Antibiotics	Macrolides (erythromycin and clarithromycin) and quinolones (ciprofloxacin, levofloxacin, and moxifloxacin)
Other drugs	Chloroquine, hydroxychloroquine, and quinine

Close and regular follow-up was planned to monitor the patient's condition and response to treatment. The family was advised to have ECGs performed on all first-degree relatives to screen for prolonged QT intervals. The case was discussed with an electrophysiologist about the potential for ICD placement due to the high risk of sudden cardiac events, although no immediate decision was made. The importance of adhering to beta-blocker therapy, avoiding QT-prolonging medications, and undergoing regular cardiac evaluations was strongly emphasized.

## Discussion

Congenital LQTSs, such as JLNS, often manifest as convulsions, syncope, or sudden cardiac arrest due to underlying dysrhythmias, leading to diagnostic delays and inappropriate treatment. A careful review of clinical and family history remains vital in detecting LQTS and JLNS, especially in pediatric emergencies or outpatient units where missed cases can have serious consequences [7]. Significant family history, such as LQTS, sudden unexplained sibling deaths, or congenital deafness, can raise clinical suspicion for JLNS. The aggressiveness of JLNS is reflected by a high mortality rate, with symptoms manifesting in 50% of patients by age three and 90% by age 18 [8].

Patients with LQTS often present with convulsions caused by transient cerebral hypoperfusion during arrhythmic events. These typically manifest as generalized convulsions, leading to frequent misdiagnosis as epilepsy [9,10]. LQTS is often misjudged in pediatric patients, and studies report initial presentations resembling seizures in up to 10% of cases. The misdiagnosis rates can reach 20-30%, which results in inappropriate anticonvulsant therapy [10,11]. This not only exposes patients to unnecessary medication risks but also delays recognition of LQTS, which increases the risk of serious complications such as sudden death [12].

Several clinical signs can help differentiate LQTS-related convulsions from epilepsy, including short convulsive episodes, often preceded by syncope, and a strong family history of LQTS or sudden unexplained death [7]. Moreover, neurological assessments, including electroencephalograms, are often normal in LQTS-induced convulsions, which serves as a valuable differentiating factor from true epilepsy [12]. Physiological QTc prolongation is common in early infancy, typically normalizing to around 400 ± 20 ms by six months of age, with no significant gender-based differences. For screening, thresholds of ≥440 ms in boys and ≥450 ms in girls are often used. Many clinicians fail to routinely measure QTc, which limits early detection [1,13]. A single ECG may overlook transient QTc changes in LQTS, whereas Holter monitoring reveals sustained abnormalities like QTc >500 ms and bradycardia, supporting diagnosis and risk stratification. Treadmill stress testing is valuable in diagnosing borderline LQTS cases and in subtype differentiation [1].

The Schwartz score combines ECG findings, clinical history, and familial risk to estimate LQTS probability. Scores below one suggest low risk, one to three intermediate, and >3.5 high probability. Despite its strong specificity (99%), the limited sensitivity (18%) means many true LQTS cases may be missed [7]. Genetic testing plays a pivotal role in confirming LQTS and is essential for risk stratification, therapeutic guidance, and comprehensive family counselling [12,13]. ECG screening is strongly recommended for populations at higher LQTS risk, including children with congenital sensorineural hearing loss, especially in communities with high consanguinity. Routine QTc measurement should be incorporated into all pediatric ECG evaluations, regardless of hearing status [1,11].

Beta-blockers remain the first-line therapy for LQTS. For high-risk patients or those unresponsive to pharmacological and lifestyle interventions, device therapies play a vital role in managing pediatric LQTS/JLNS, but their implementation can cause significant distress, raising ethical concerns that demand a tailored, multidisciplinary approach [1,14]. Early ECG evaluation, Schwartz scoring, and genetic testing are vital to prevent sudden death and improve patient outcomes [7,10].

## Conclusions

This case highlights the importance of considering JLNS in children having convulsions, syncope, or congenital hearing loss, especially when epilepsy treatment fails and there is a relevant family history. Every child being evaluated for seizures must undergo a standard 12-lead ECG along with a QT interval measurement. Early recognition through ECG and clinical correlation can be life-saving, even in the absence of genetic confirmation. Initiating appropriate therapy with beta-blockers, avoiding known triggers, and involving a multidisciplinary team are critical steps in reducing the risk of sudden cardiac death. This report also emphasizes the importance of awareness and clinical suspicion in resource-limited areas, where advanced diagnostics are unavailable.
